# Combined resistance exercise and essential amino acid intake enhance follistatin/myostatin ratio and muscle fitness in older women: a randomized controlled trial

**DOI:** 10.1080/15502783.2026.2646626

**Published:** 2026-03-21

**Authors:** Deokhwa Jeong, Rudy J. Valentine, Hyeongmo Jeong, Jun-Young Sung, Heekyoung Lim, Sunghwun Kang

**Affiliations:** a Department of Smart Health Science and Technology, Kangwon National University, Gangwon-do, Republic of Korea; b Department of Physical Therapy & Kinesiology, University of Massachusetts Lowell, Lowell, MA, United States; c Department of Sport Science, Kangwon National University, Gangwon-do, Republic of Korea; d Institute of Sports Medicine & Nutrition, Kwangwoon University, Seoul, Republic of Korea; e Querencia, a Forest Walfare Business, Gangwon-do, Republic of Korea

**Keywords:** Resistance exercise, essential amino acid, myokines, muscle fitness, muscle mass

## Abstract

**Background:**

Age-associated sarcopenia and declining physical function in older women are connected to changes in hormones, inflammation, and disrupted protein metabolism. Myokines and cytokines play central roles in muscle atrophy. While both resistance exercise (RE) and essential amino acid (EAA) supplementation are promising interventions, limited randomized trials have assessed their combined effect in healthy elderly populations. Early targeted strategies may help delay sarcopenia and promote healthier aging.

**Methods:**

A 12-week randomized controlled trial was performed involving 96 healthy women aged ≥ 65 years without insulin resistance. Participants were randomized into four groups: control, RE, EAA, or RE + EAA. The intervention consisted of a circuit-based training program conducted three times per week, with each session lasting 60 minutes at moderate intensity. Participants in EAA and RE + EAA groups consumed 5.5g of EAA twice daily. Assessments before and after the intervention included body composition, muscle fitness, serum myokines, and inflammatory cytokines. Data analysis involved two-way repeated measures ANOVA, Bonferroni post-hoc comparisons, and one-way ANOVA for changes in the follistatin/myostatin ratio.

**Results:**

The RE + EAA group demonstrated a significant increase in muscle mass (F(3, 72) = 5.042, *p* < 0.001, partial η² = 0.174) and greater improvements in the senior fitness test (*p* ranging from < 0.05 to < 0.001). There was a reduction in myostatin levels (*p* < 0.05) and an elevation in follistatin in both the RE (*p* < 0.05) and RE + EAA (*p* < 0.001) groups. The follistatin/myostatin ratio increased most in the RE + EAA group (F(3, 72) = 5.556, *p* = 0.002, partial η² = 0.188), with significance versus control (*p* < 0.001), EAA (*p* < 0.05) groups. IL-6 and IL-1β were significantly reduced in the RE (*p* < 0.05) and RE + EAA (*p* < 0.05) groups, whereas TNF-*α* decreased only in the RE + EAA group (*p* < 0.05).

**Conclusion:**

A 12-week intervention combining resistance exercise and essential amino acid supplementation was superior to either intervention alone in enhancing muscle mass, muscle fitness, myokine profiles, and reducing inflammatory markers among healthy older women. These results support the development of early combined interventions for the prevention of sarcopenia and may guide personalized exercise-nutrition prescriptions for optimal aging.

**Trial registration:**

KCT 0010756 (Retrospectively registered; July 15, 2025).

## 1. Introduction

Age-related biological changes generally have more adverse than beneficial consequences. Throughout a woman’s life following puberty, hormonal fluctuations persist, with menopause marking an abrupt hormonal transition due to aging that produces significant negative impacts not only on body composition but also on metabolic health [1]. Specifically, after menopause, women experience reduced estrogen levels, which can promote visceral adiposity, metabolic disturbances such as insulin resistance, increased type 2 diabetes risk, and impaired regulation of skeletal muscle protein synthesis and breakdown. Such dysregulation leads to the loss of muscle mass and impaired physical capacity [2].

The secretion of myokines by skeletal muscle is essential for preserving muscle mass and regulating metabolic functions [3]. Myostatin, classified as a myokine, serves as a negative regulator of muscle growth by suppressing the proliferation and differentiation of satellite cells, ultimately leading to muscle atrophy [4]. Conversely, follistatin directly antagonises myostatin activity, facilitating muscle growth and hypertrophy by functioning as a potent anabolic factor [5]. Given their contrasting functions, the ratio of follistatin to myostatin (F/M ratio) is recognised as a significant physiological marker that reflects the homeostasis between anabolic and catabolic activities in skeletal muscle [4]. It is regarded as a sensitive biomarker for assessing muscle health and function [5,6]. Additionally, age-related chronic low-grade inflammation involves increased pro-inflammatory cytokines, such as TNF-*α* and IL-6, which are known to contribute to muscle wasting and reduced physical function [7].

Within this context, resistance exercise (RE) and essential amino acid (EAA) supplementation are increasingly recognised as leading non-pharmacological strategies for mitigating sarcopenia, a prevalent age-related condition. RE imposes mechanical stimuli on skeletal muscle, thereby activating the mTORC1 pathway and enhancing muscle protein synthesis (MPS), which is vital for preserving muscle mass [8,9]. Supplementation with EAA, particularly those enriched with leucine, has been identified as an effective nutritional intervention to address anabolic resistance, a common decline in the efficiency of protein synthesis with advancing age, thus supporting muscle metabolic processes [10,11]. Emerging evidence indicates that both RE and EAA independently support improvements in muscle mass and function by modulating myostatin, follistatin, and pro-inflammatory cytokines [12,13]. Furthermore, concurrent RE and EAA interventions are being explored in clinical settings for their potential to produce enhanced and synergistic benefits in muscle mass and functional performance compared to the effects of either approach alone [14].

However, most available evidence originates from animal studies or research on older adults with metabolic disorders [15,16], highlighting a substantial gap in clinical studies examining the combined effects of RE and EAA in healthy older women. Although insulin resistance and metabolic syndrome are recognised as primary contributors to the progression of sarcopenia, research on early preventive strategies remains limited. Sarcopenia serves as an early indicator of age-associated physiological deterioration, and interventions during its onset may slow functional decline and avert chronic conditions. Thus, investigating the co-effects of RE and EAA in healthy older adults could generate important data to guide early intervention strategies for healthy aging.

Therefore, this study is designed to assess the effects of a 12-week program involving RE, EAA supplementation, and their combination, on muscle mass, muscle function, key myokines, and pro-inflammatory cytokines in healthy women aged 65 years and older who do not have insulin resistance, utilising a randomized controlled trial (RCT) framework. This research aims to generate scientific evidence supporting effective, non-pharmacological preventive strategies for sarcopenia, and to enhance physical function among healthy older adults, thereby facilitating the integration of targeted approaches for advancing healthy aging.

## 2. Methods

### 2.1. Study design & participation

The study adhered to the Declaration of Helsinki and was approved by the Institutional Review Board of Kangwon National University (KWNUIRB-2024-11-006). It was retrospectively registered in the Korea Clinical Trials Registry (KCT0010756; July 15, 2025). Elderly women (72.8 ± 4.57 years) participated in the study. Written informed consent was secured from all participants prior to enrolment. Sample size was calculated using G*Power software (version 3.1.9.7; Heinrich Heine University) with an assumed medium effect size (f = 0.25), *α* = 0.05, and statistical power (1–β) = 0.95. The minimum required sample size was determined to be 76. Anticipating a dropout rate of 20%, more than 91 individuals were initially recruited.

Participants were screened using predefined criteria. Inclusion criteria specified that participants must be physically capable of performing resistance exercise, able to consume essential amino acids, and willing to participate voluntarily. Exclusion criteria were as follows: (1) diagnosis of a musculoskeletal disorder within the previous 6 months that limited activity; (2) severe cardiovascular disease precluding resistance exercise; (3) inability to perform daily activities independently due to depression, anxiety, or insomnia; (4) current use of glucose-lowering medications; (5) engagement in dieting or structured exercise involving ≥ 2 planned sessions/week; and (6) any condition, as determined by investigators or attending physicians, deemed likely to compromise study validity. Use of stable antihypertensive or lipid-lowering medications was permitted.

Before baseline assessments, fasting glucose and fasting insulin levels were obtained, and insulin resistance was evaluated using the homeostasis model assessment of insulin resistance (HOMA-IR): (fasting insulin [μIU/mL] × fasting glucose [mmol/L])/22.5 [17]. Participants with HOMA-IR > 2.5 were excluded because previous studies have demonstrated that elevated insulin resistance impairs anabolic signaling, decreases muscle protein synthesis, and alters exercise and amino acid–induced biomarker responses [18]. Including metabolically dysregulated participants could have confounded the interpretation of post-intervention effects and reduced the internal validity of the study. Out of 130 individuals initially assessed, 5 withdrew prior to baseline screening, and 29 were excluded due to HOMA-IR values greater than 2.5. Ultimately, 96 participants met eligibility criteria and were enrolled.

**Figure 1.**
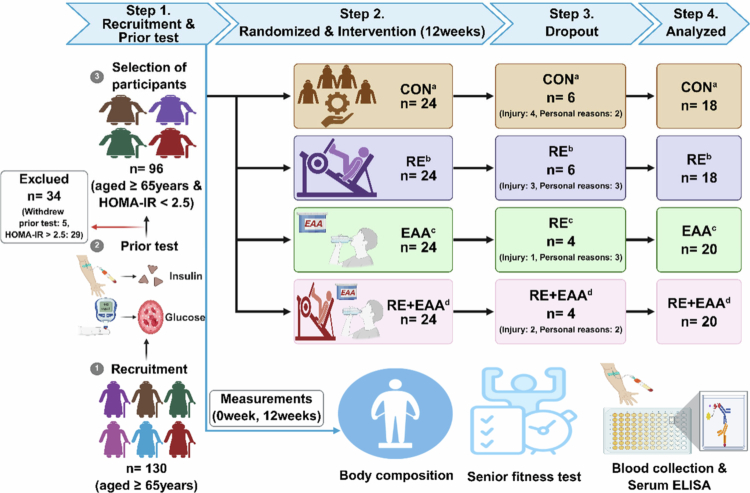
Overview of study design. Study design. CON^a^; control group, RE^b^; resistance exercise group, EAA^c^; essential amino acid intake group, RE+EAA^d^; resistance exercise and essential amino acid intake group. The intervention consisted of resistance exercise three times weekly (60 min per session) and essential amino acid supplementation twice daily (5.5 g each time).

The study design and initial participant testing results are presented in Figure 1 and Table 1.

**Table 1.** Assessment of prior risk factors for insulin resistance.

Variable	Included (*n* = 96)	Excluded (*n* = 29)
Age	73.17 ± 3.87	73.06 ± 5.22
Glucose(mmol/L)	7.72 ± 1.17	8.23 ± 0.98
Insulin (μIU/ml)	4.48 ± 1.95	9.76 ± 2.96
HOMA-IR	1.52 ± 0.76	3.57 ± 1.20

Reported values represent the Mean ± SD. Inclusion criteria: HOMA-IR < 2.5; Exclusion criteria: HOMA-IR > 2.5. HOMA-IR refers to the Homoeostatic Model Assessment of Insulin Resistance, calculated as [Fasting insulin (μIU/ml) × Fasting blood glucose (mmol/L)] ÷ 22.5 [17].

All participants completed baseline (Pre) and 12 weeks post-intervention (Post) assessments at the Exercise Physiology Laboratory, Department of Sports Science, Kangwon National University. All evaluations were conducted following an overnight fast. Participants were randomly assigned at baseline to one of four groups using opaque envelopes labelled with group names. The four groups were: control group (CON), resistance exercise group (RE), essential amino acid intake group (EAA), and combined RE and EAA intake group (RE + EAA), with 24 participants each. During the intervention period, 20 participants dropped out: 6 from the CON, 6 from the RE, 4 from the EAA, and 4 from the RE + EAA. Thus, 76 participants completed the study.

**Figure 2.**
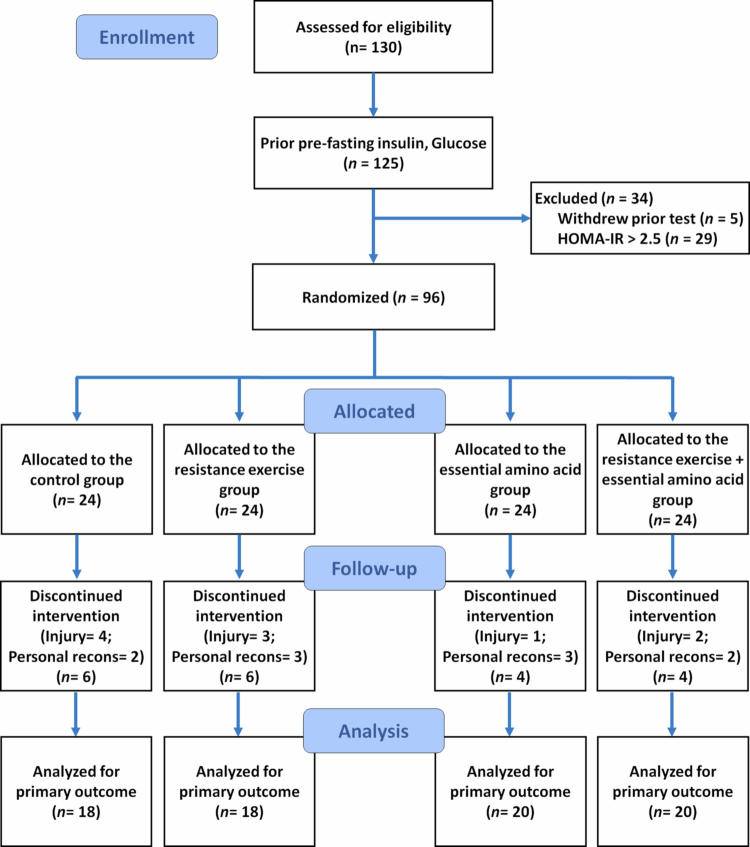
CONSORT participant flow diagram.

The CONSORT flow diagram for participant progression is shown in Figure 2.

### 2.2. Haematological analysis

Venous blood samples were collected from all participants at baseline and again at 12 weeks following treatment. Fasting was maintained for 12 h after the evening meal on the previous day. Blood samples were drawn the next morning after adequate rest, minimizing physical activity. All samples were performed at 08:00 from the antecubital veins. All venipuncture procedures were performed by a licensed nurse. To control for acute effects, all samples were collected at last 48 hours after the last exercise session and the most recent EAA intake. Whole blood was collected in serum tubes, clot at room temperature for 30 minutes, and centrifuged at 3,500g for 10 minutes. The resulting serum samples were preserved at -80 °C until further analysis. Fasting glucose levels were assessed using the One Touch Ultra system (LifeScan), based on glucose oxidase chemistry. Serum concentrations of fasting insulin (440132, Beckman Coulter, California, CA, USA), serum levels of myokine biomarkers including IL-15, irisin, myostatin, and follistatin (DY247, DY9420-05, DY788-05, and DY669: R&D Systems, Minneapolis, MN, USA), and inflammatory cytokine biomarkers such as IL-1β, IL-6, and tumour necrosis factor-alpha (TNF-*α*) (DY201, DY206, and DY210: R&D Systems, Minneapolis, MN, USA) were measured using DuoSet TM enzyme-linked immunosorbent assay (ELISA) kits (R&D Systems, Minneapolis, MN, USA) in accordance with the manufacturer’s instructions.

### 2.3. Assessment of body composition

Body composition variables were measured using a bioelectrical impedance analyser (BIA) (Inbody 720 Body Composition Analyser, Biospace, Seoul, Republic of Korea). Participants were assessed barefoot and in light clothing, after removing shoes, socks, and heavy accessories. Weight (kg), fat mass (kg), and skeletal muscle mass (kg) were recorded to the nearest 0.1 kg. Body fat percentage was calculated based on impedance values obtained through multiple frequencies. Body mass index (BMI) was determined as weight in kilograms divided by height in metres squared (kg/m²).

### 2.4. Assessment of the Senior Fitness Test

Physical fitness was measured using the Senior Fitness Test (SFT) developed and validated by Rikli and Jones [19]. All participants completed the Senior Fitness Test (SFT) at baseline and after 12 weeks. The SFT evaluated six components: muscle strength, muscular endurance, flexibility, balance, cardiorespiratory endurance, and coordination. Muscle strength was measured using a digital hand grip dynamometer (BS-HG, Biospace, Korea). Grip strength was tested twice per hand, and the average of the two highest values (one per hand) was used. Relative grip strength was computed according to the following formula: [(highest right-hand grip + highest left-hand grip) ÷ 2] × 100 ÷ body weight (kg). Muscular endurance was assessed via the 30-second chair stand test using a 40 cm chair, where participants crossed their arms over the chest and repeatedly stood up and sat down completely for 30 seconds. Flexibility was evaluated using a sit and reach procedure with a digital forward flexion meter (BS-FF, Biospace, Korea). Balance was assessed via the Timed Up and Go (TUG) test, which involved rising from a chair, walking 3 meters around a cone, and returning to sit. Coordination was assessed using the Figure of 8 Walk Test (F8WT), performed on a rectangular course measuring 3.6 m by 1.6 m, with cones placed at each corner and two additional cones diagonally 2.4m apart. Participants began seated, stood up, walked around the rear right cone, returned to sit, and then repeated the same for the rear left cone twice, with total completion time recorded. Cardiorespiratory endurance was measured using the 2-minute step test, in which participants marched in place for 2 minutes, lifting the knees to a height midway between the patella and the iliac crest; only steps meeting this height were counted. Strength and flexibility tests were performed twice, and the higher value was recorded. All SFT evaluations were administered by a certified health and exercise specialist.

### 2.5. Essential amino acid intake

Participants in both the EAA and RE + EAA groups received an essential amino acid supplement (EAALPHA, Prinova Group LLC, Itasca, IL, USA) containing 5.5 g of EAA per serving, taken twice daily. EAA ingestion occurred within 30 minutes post-exercise and between meals. Participants were instructed to dissolve one packet of EAA in plain water and consume it twice daily for 12 weeks. On days involving participation in the exercise programme, intake was scheduled after exercise completion. On non-exercise days, intake timing was permitted at the participant's discretion. Throughout the 12-week period, participant compliance with EAA consumption was carefully monitored and supported. EAA supplements were distributed weekly, providing a seven-day supply (14 sachets per week). Participants were reminded daily via group text messages to confirm intake and maintain adherence. While compliance was not quantified as numerical data, all participants provided written consent to take two servings of EAA per day and reported full compliance throughout the intervention. Our research team verified adherence through continuous communication and weekly collection checks, ensuring that all participants followed the prescribed intake protocol in an ethically verifiable manner.

The dosage (5.5 g per serving; 1.4 g leucine) was selected based on prior evidence suggesting that approximately 10 g of EAA can stimulate muscle protein synthesis in older adults despite age-related anabolic resistance [20]. However, it should be noted that the study by Cuthbertson et al. demonstrated a maximal anabolic response following a single bolus ingestion of ~10 g EAA rather than across a total daily dosing regimen. In the present study, EAA was administered in divided doses (5.5 g twice daily), and therefore, direct extrapolation from single-dose response data should be interpreted with caution. Indeed, lower single doses (~5 g) have been reported to produce submaximal anabolic responses, representing a potential limitation when translating mechanistic findings to daily supplementation protocols. Nevertheless, the selected formulation was considered physiologically appropriate and practical for the present population.

The constituent of the EAA supplement is detailed in Table 2.

**Table 2.** Constituents of EAA.

Variable	Total: 5500 mg
Sodium	40 mg
Carbohydrate	1000 mg
Fat	100 mg
L-Leucine	1389 mg
L-Valine	357 mg
L-Arginine	321 mg
L-Phenylalanine	218 mg
L-Histidine	53 mg
L-Isoleucine	347 mg
L-Threonine	304 mg
L-Methionine	107 mg
L-Lysine Monohydrochloride	562 mg
L-Tryptophan	2 mg
Flavouring agent	700 mg

### 2.6. Exercise intervention

The resistance exercise programme was based on established protocols [21], with adjustments for older adults’ physiological characteristics. It adhered to American College of Sports Medicine (ACSM) and National Strength and Conditioning Association (NSCA) guidelines for older adults [22]. Only the RE and RE + EAA groups performed a circuit-style resistance training programme that targeted major muscle groups, including the chest, back, arms, and legs. Training sessions were held three times weekly, using resistance bands (weeks 1–6) with progressive tension, followed by kettlebells (weeks 7–12) to increase intensity. All sessions were supervised by certified exercise professionals. Each session included 4–6 exercises, with difficulty tailored to individual fitness levels. To promote progressive overload while minimising risk, exercises were structured into four levels of difficulty. During the initial phase, participants performed one set of 10–15 repetitions per exercise, focusing on proper technique. Training volume was then systematically increased every three weeks by adjusting both repetitions and the number of sets. Sets increased from one to three, followed by higher repetition targets (15–20 reps) as tolerated. Rest intervals of 1–3 minutes were provided between sets. Before the intervention, each participant’s initial resistance level was determined using bands that allowed 10–15 repetitions with proper form. During weeks 1–3, all participants used red bands (tension 3.7 lbs), followed by green bands (tension 4.6 lbs) during weeks 4–6. From week 7 onward, exercises were performed using kettlebells starting at 6–8 kg, which were progressively increased to 10–12 kg from week 10. Progressive tension and load adjustments were made based on individual tolerance and performance capacity to ensure continuous overload and participant safety. Exercise intensity and corresponding energy expenditure were objectively tracked using a heart rate sensor (Polar H10, Polar Electro Oy, Finland). The intended energy expenditure for each session was approximately 250-350 kcal, based on individual baseline assessments. The Polar H10 sensor has been validated as a reliable and accurate tool for measuring heart rate and estimating exercise-related energy expenditure when compared with electrocardiogram (ECG) and indirect calorimetry systems [23,24]. Intensity was adjusted every three weeks to maintain challenge.

Detailed information regarding the exercise intervention can be found in Table 3.

**Table 3.** Resistance exercise protocol.

Variable parameter	Resistance training protocol			Duration (min)	Intensity	Frequency
Warm up	Dynamic Stretching			10	Band exercise 1 ∼ 3 weeks 11 ∼ 12 RPE 4 ∼ 6 weeks 12 ∼ 14 RPE 250 ∼ 300 Kcal	12 weeks (3 times per week) 36 sessions
Main Exercise	Monday (Upper body)	Wednesday (Lower body)	Friday (Combination)	40		
	Chest press	Squat	Thruster			
	Bent-over row	Wide squat	Dead lift & Bent over row			
					Kettlebell exercise 7 ∼ 9 weeks 14 ∼ 15 RPE 9 ∼ 12 weeks 14 ∼ 15 RPE 300 ∼ 350 Kcal	
	Overhead press	Side tap	Squat & Calf raise			
	Triceps extension	Leg press	Press & Squat			
	Biceps curl	Hip abduction	Side tap & Squat			
Cool down	Static Stretching			10	HRmax 60 ∼ 80%	

### 2.7. Control lifestyle

All participants were instructed to maintain their habitual daily routines and to avoid initiating any new structured exercise that could potentially impact the study outcomes. Participants were also requested not to implement major dietary modifications that might influence body composition. Although no specific software was used to record dietary intake or daily physical activity, compliance was monitored through weekly self-reports and regular group text message reminders throughout the intervention period to minimize potential confounding effects. Additionally, all participants signed a written agreement confirming that they would not participate in any other exercise, weight loss, or health-related programmes that could influence the study outcomes, and that they would adhere to the investigators’ instructions throughout the trial.

### 2.8. Statistical analysis

All results are presented as mean ± standard deviation. Statistical analyses were performed with SPSS version 29.0 (SPSS Inc., Chicago, IL, USA). An initial two-way repeated measures analysis of variance (ANOVA) was conducted to assess the main effects of group, time, and their interaction. If significant interaction effects were found, post-hoc analyses involved Bonferroni-adjusted pairwise comparisons for between-group differences and paired-sample t-tests to compare within-group differences over time. All statistical tests were two-tailed, and effect sizes were calculated as partial η² to indicate the magnitude of observed effects. Statistical significance was defined as *α* = 0.05. Non-significant trends are described up to *p* = 0.1 in the text. Furthermore, between-group differences in the percentage change of the follistatin/myostatin ratio were evaluated using one-way ANOVA, with Bonferroni post-hoc tests applied to determine which comparisons differed significantly.

## 3. Results

### 3.1. Change in body composition

Results for body composition parameters are shown in Table 4. Two-way repeated-measures ANOVA demonstrated significant group-by-time interaction effects for muscle mass (F(3, 72) = 5.042, *p* = 0.003, partial η² = 0.174) and BMI (F(3, 72) = 3.248, *p* = 0.027, partial η² = 0.119). There were significant main effects of time on weight (F(1, 72) = 13.884, *p* < 0.001, partial η² = 0.162), muscle mass (F(1, 72) = 5.572, *p* = 0.021, partial η² = 0.072), fat mass (F(1, 72) = 56.400, *p* < 0.001, partial η² = 0.439), body fat percentage (F(1, 72) = 44.747, *p* < 0.001, partial η² = 0.383), and BMI (F(1, 72) = 8.446, *p* = 0.005, partial η² = 0.105). The main effect of group was not significant for any variable (all *p* > 0.05). Paired t-tests showed that the RE + EAA group had a significant increase in muscle mass after 12 weeks relative to baseline (*p* < 0.001). Significant reductions in weight were found in the CON and RE groups (*p* < 0.05). Fat mass and body fat percentage were significantly reduced after 12 weeks in the RE, EAA, and RE + EAA groups (*p* < 0.05 or *p* < 0.001). BMI decreased significantly in the CON and RE groups (*p* < 0.05). Waist circumference did not show significant changes in any group (F(3, 72) = 1.706, *p* = 0.173, partial η² = 0.066).

**Table 4.** Effects on body composition.

Variable	Group	Baseline (0 week)	Post (12 weeks)	F-Value (*p*-Value/Partial η²)	Post-Hoc
Age	CON (*n* = 18)^a^	74.11 ± 2.35		N/S	N/S
	RE (*n* = 18)^b^	72.89 ± 4.89			
	EAA (*n* = 20)^c^	72.80 ± 4.57			
	RE + EAA(*n* = 20)^d^	72.95 ± 2.54			
Height (cm)	CON (*n* = 18)^a^	151.24 ± 5.14	151.27 ± 4.64	G: 0.591 (0.623/0.024) T: 0.174 (0.677/0.002) G × T: 1.546 (0.210/0.061)	N/S
	RE (*n* = 18)^b^	152.39 ± 5.36	153.26 ± 4.91		
	EAA (*n* = 20)^c^	152.81 ± 4.74	152.95 ± 4.40		
	RE + EAA (*n* = 20)^d^	153.17 ± 3.17	152.54 ± 3.98		
Weight (kg)	**CON (*n* = 18)** ^**a**^	**55.41 ± 6.81**	**54.70 ± 7.00** ^*^	G: 0.831 (0.481/0.033) **T: 13.884 (<0.001/0.162)** **G × T: 3.135 (0.031/0.116)**	N/S
	**RE (*n* = 18)** ^**b**^	**57.39 ± 8.67**	**56.68 ± 8.26** ^*^		
	EAA (*n* = 20)^c^	58.84 ± 8.14	58.83 ± 8.34		
	RE + EAA (*n* = 20)^d^	57.35 ± 5.91	57.22 ± 5.56		
Muscle mass (kg)	CON (*n* = 18)^a^	22.94 ± 3.27	22.82 ± 3.57	G: 1.001 (0.398/0.041) **T: 5.572 (0.021/0.072)** **G × T: 5.042 (0.003/0.174)**	N/S
	RE (*n* = 18)^b^	22.67 ± 1.86	22.93 ± 1.94		
	EAA (*n* = 20)^c^	23.10 ± 3.61	23.05 ± 3.21		
	**RE + EAA (*n* = 20)** ^**d**^	**21.29 ± 2.01**	**22.05 ± 2.30** ^***^		
Fat mass (kg)	CON (*n* = 18)^a^	17.94 ± 2.42	17.39 ± 2.38	G: 1.182 (.323/0.047) **T: 56.400 (<0.001/0.439)** G × T: 1.084 (0.362/0.043)	N/S
	**RE (*n* = 18)** ^**b**^	**19.43 ± 2.45**	**18.34 ± 2.21** ^***^		
	**EAA (*n* = 20)** ^**c**^	**20.48 ± 6.15**	**19.37 ± 5.74** ^***^		
	**RE + EAA(*n* = 20)** ^**d**^	**18.92 ± 3.27**	**17.87 ± 3.26** ^***^		
Body fat (%)	CON (*n* = 18)^a^	32.49 ± 3.10	31.95 ± 3.50	G: .714 (0.547/0.029) **T: 44.747 (<0.001/0.383)** G × T: 1.955 (0.128/0.075)	N/S
	**RE (*n* = 18)** ^**b**^	**34.23 ± 3.07**	**32.59 ± 2.84** ^***^		
	**EAA (*n* = 20)** ^**c**^	**34.21 ± 5.57**	**32.38 ± 4.96** ^*^		
	**RE + EAA(*n* = 20)** ^**d**^	**32.88 ± 3.31**	**31.01 ± 3.59** ^***^		
BMI (kg/m^2^)	**CON (*n* = 18)** ^**a**^	**18.32 ± 2.15**	**18.08 ± 2.24** ^*^	G: .685 (.564/0.028) **T: 8.446 (0.005/0.105)** **G × T: 3.248 (0.027/0.119)**	N/S
	**RE (*n* = 18)** ^**b**^	**18.74 ± 2.65**	**18.46 ± 2.36** ^*^		
	EAA (*n* = 20)^c^	19.24 ± 2.50	19.21 ± 2.53		
	RE + EAA (*n* = 20)^d^	18.71 ± 1.75	18.75 ± 1.65		
Waist (cm)	CON (*n* = 18)^a^	82.75 ± 7.62	81.28 ± 7.27	G: 1.999 (0.122/0.077) T: 2.127 (0.149/0.029) G × T: 1.706 (0.173/0.066)	N/S
	RE (*n* = 18)^b^	84.03 ± 7.91	83.21 ± 8.28		
	EAA (*n* = 20)^c^	83.06 ± 8.55	83.67 ± 8.02		
	RE + EAA (*n* = 20)^d^	78.78 ± 5.09	78.50 ± 5.18		

Values are presented as Mean ± SD. CON, control group; RE, resistance exercise; EAA, essential amino acid intake group; RE + EAA, resistance exercise and essential amino acid intake group. Statistical analysis was performed using the paired t-test: *p < 0.05; ***p < 0.001. Effect sizes (partial η²) were interpreted as small (0.01–0.059), medium (0.06–0.139), and large (≥0.14).

### 3.2. Change in Senior Fitness Test

Results for senior fitness test parameters are shown in Table 5. Two-way repeated-measures ANOVA demonstrated significant group-by-time interaction effects for the 30-sec chair stand (F(3, 72) = 2.834, *p* = 0.044, partial η² = 0.106), 2-minute step (F(3, 72) = 5.083, *p* = 0.003, partial η² = 0.175), Timed Up & Go (F(3, 72) = 5.911, *p* = 0.001, partial η² = 0.198), and F8WT (F(3, 72) = 5.601, *p* = 0.002, partial η² = 0.189). Significant main effects of time were observed for relative grip strength (F(1, 72) = 11.807, *p* < 0.001, partial η² = 0.141), 30-sec chair stand (F(1, 72) = 22.677, *p* < 0.001, partial η² = 0.240), 2-minute step (F(1, 72) = 22.234, *p* < 0.001, partial η² = 0.236), sit & reach (F(1, 72) = 6.712, *p* = 0.012, partial η² = 0.085), Timed Up & Go (F(1, 72) = 27.551, *p* < 0.001, partial η² = 0.277), and Figure-of-8 Walk Test (F(1, 72) = 65.096, *p* < 0.001, partial η² = 0.475). The main effect of group was significant only for the 2-minute step (F(3, 72) = 7.314, *p* < 0.001, partial η² = 0.234) and F8WT (F(3, 72) = 5.348, *p* = 0.002, partial η² = 0.182). Paired t-tests showed that the RE group exhibited significant improvements from baseline to post-intervention in relative grip strength (*p* < 0.05), 30-sec chair stand (*p* < 0.05), 2-minute step (*p* < 0.001), sit & reach (*p* < 0.05), Timed Up & Go (*p* < 0.001), and Figure-of-8 Walk Test (*p* < 0.001). The RE + EAA group demonstrated significant improvements in the 30-sec chair stand (*p* < 0.05), 2-minute step (*p* < 0.001), Timed Up & Go (*p* < 0.05), and Figure-of-8 Walk Test (*p* < 0.05). In the EAA group, significant improvement was observed only in the 30-sec chair stand (*p* < 0.05) and F8WT (*p* < 0.05).

**Table 5.** Senior fitness test outcome measures.

Variable	Group	Baseline (0 week)	Post (12 weeks)	F-Value (*p*-Value/Partial η²)	Post-Hoc
Relative grip Strength (%BW)	CON (*n* = 18)^a^	39.45 ± 6.13	39.92 ± 6.43	G: 0.309(.0.819/0.013) **T: 11.807(<0.001/0.141)** G × T: 1.836(0.148/0.071)	N/S
	**RE (*n* = 18)** ^**b**^	**39.38 ± 7.95**	**42.48 ± 6.44** ^*^		
	EAA (*n* = 20)^c^	39.96 ± 6.28	40.57 ± 5.69		
	**RE + EAA(*n* = 20)** ^**d**^	**38.05 ± 6.01**	**40.18 ± 6.30** ^*^		
30 sec Chair Stand (rep)	CON (*n* = 18)^a^	16.61 ± 3.48	17.72 ± 4.62	**G: 12.981(<0.001/0.351)** **T: 22.677(<0.001/0.240)** **G × T: 2.834(0.044/0.106)**	G: a < b, c, d G × T: a < b, c, d
	**RE (*n* = 18)** ^**b**^	**20.72 ± 5.85**	**26.00 ± 7.18** ^*^		
	EAA (*n* = 20)^c^	22.70 ± 5.67	23.95 ± 4.52		
	**RE + EAA (*n* = 20)** ^**d**^	**23.75 ± 4.67**	**28.10 ± 5.12** ^*^		
2 min Step (rep)	CON (*n* = 18)^a^	97.06 ± 12.75	99.72 ± 18.18	**G: 7.314(<0.001/0.234)** **T: 22.234 (<0.001/0.236)** **G × T: 5.083 (0.003/0.175)**	G: a < c, d G × T: a < b, c, d
	**RE (*n* = 18)** ^**b**^	**99.17 ± 15.84**	**120.22 ± 15.50** ^***^		
	EAA (*n* = 20)^c^	116.65 ± 27.46	119.60 ± 14.59		
	**RE + EAA (*n* = 20)** ^**d**^	**113.35 ± 11.08**	**122.05 ± 13.87** ^***^		
Sit & Reach (cm)	CON (*n* = 18)^a^	12.14 ± 6.58	12.26 ± 6.55	G: .1.281(0.287/0.051) **T: 6.712 (0.012/0.085)** G × T: 2.276 (0.087/0.087)	N/S
	**RE (*n* = 18)** ^**b**^	**8.65 ± 7.55**	**10.65 ± 7.24** ^*^		
	EAA (*n* = 20)^c^	13.32 ± 9.01	13.21 ± 9.64		
	RE + EAA (*n* = 20)^d^	13.75 ± 9.64	15.49 ± 8.25		
Timed Up & Go (TUG) (sec)	**CON (*n* = 18)** ^**a**^	**7.11 ± 1.05**	**6.41 ± 1.23** ^*^	**G: 5.112(0.003/0.176)** **T: 27.551 (<0.001/0.227)** **G × T: 5.911 (0.001/0.198)**	G: d < a G × T: d < a, c
	**RE (*n* = 18)** ^**b**^	**6.55 ± 1.23**	**5.59 ± 1.13** ^***^		
	EAA (*n* = 20)^c^	6.46 ± 1.06	6.48 ± 1.19		
	RE + EAA (*n* = 20)^d^	5.76 ± 0.57	5.50 ± 0.53		
Figure of 8 Walk Test (F8WT) (sec)	**CON (*n* = 18)** ^**a**^	**29.78 ± 5.42**	**25.86 ± 5.89** ^***^	**G: 5.348(0.002/0.182)** **T: 65.096 (<0.001/0.475)** **G × T: 5.601 (0.002/0.189)**	G: d < a G × T: d < a, c
	**RE (*n* = 18)** ^**b**^	**27.59 ± 8.68**	**22.88 ± 6.18** ^***^		
	**EAA (*n* = 20)** ^**c**^	**27.08 ± 4.88**	**25.65 ± 4.16** ^*^		
	**RE + EAA (*n* = 20)** ^**d**^	**22.36 ± 2.30**	**20.91 ± 2.03** ^*^		

Values are presented as Mean ± SD. CON, control group; RE, resistance exercise; EAA, essential amino acid intake group; RE+EAA, resistance exercise and essential amino acid intake group. Statistical analysis was performed using the paired t-test: *p < 0.05; ***p < 0.001. Effect sizes (partial η²) were interpreted as small (0.01–0.059), medium (0.06–0.139), and large (≥0.14).

### 3.3. Change in myokine factors

**Figure 3.**
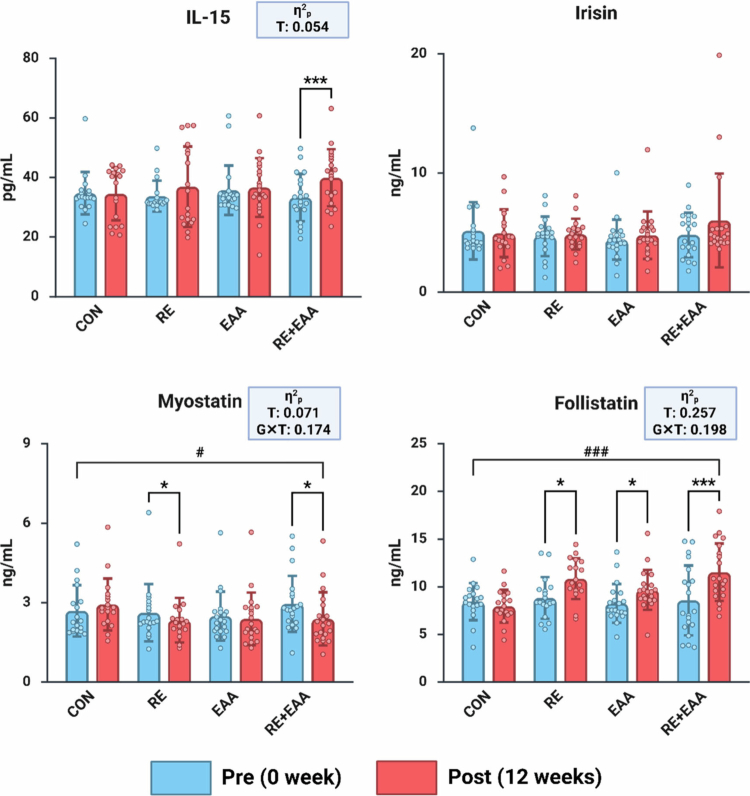
Alterations in myokine factors. Values are reported as Mean ± SD. CON, control group; RE, resistance exercise group; EAA, essential amino acid intake group; RE+EAA, combined resistance exercise and essential amino acid intake group. Statistical analysis was conducted using two-way repeated measures ANOVA. Interaction effect: ^#^*p* < 0.05, ^###^*p* < 0.001; paired t-test: *p < 0.05; ***p < 0.001. Effect sizes (η²p) were interpreted as small (0.01–0.059), medium (0.06–0.139), and large (≥0.14).

Figure 3 shows changes in myokine factor. Two-way repeated-measures ANOVA identified significant group-by-time interaction effects for myostatin (F(3, 72) = 5.040, *p* = 0.003, partial η² = 0.174) and follistatin (F(3, 72) = 5.920, *p* = 0.001, partial η² = 0.198). Significant main effects of time were also found for IL-15 (F(1, 72) = 4.105, *p* = 0.046, partial η² = 0.054), myostatin (F(1, 72) = 5.482, *p* = 0.022, partial η² = 0.071), and follistatin (F(1, 72) = 24.857, *p* < 0.001, partial η² = 0.257). According to paired t-tests, both the RE group (*p* < 0.05) and the RE + EAA group (*p* < 0.05) demonstrated significant decreases in myostatin after 12 weeks compared with baseline. Significant increases in follistatin were observed in the RE group (*p* < 0.05), the EAA group (*p* < 0.05), and the RE + EAA group (*p* < 0.001) after 12 weeks of intervention. In addition, IL-15 levels showed a tendency to increase following the intervention, as observed in the RE + EAA group (*p* < 0.001).

### 3.4. Change in Follistatin/Myostatin ratio

**Figure 4.**
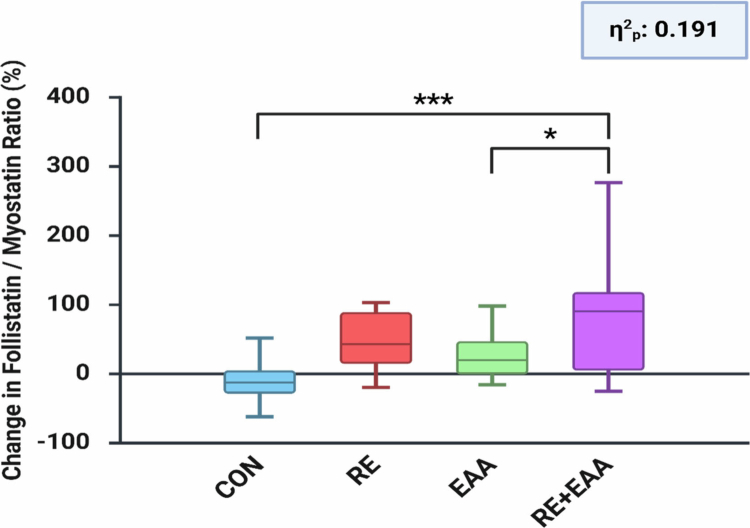
Alterations in the follistatin/myostatin ratio. The follistatin/myostatin ratio was determined based on pre- and post-intervention levels for each biomarker. Percentage changes in the ratio were determined using the following formula: [(Post follistatin / Post myostatin) / (Pre follistatin / Pre myostatin) − 1] × 100. Differences among groups were evaluated with one-way ANOVA followed by a Bonferroni post hoc test: *p < 0.05; ***p < 0.001. Effect sizes (η²p) were interpreted as small (0.01–0.059), medium (0.06–0.139), and large (≥0.14).

Figure 4 shows the percentage change in the follistatin/myostatin ratio after 12 weeks of intervention. One-way ANOVA revealed significant group differences (F(3, 72) = 5.652, *p* = 0.002, partial η² = 0.191), indicating a large effect size according to conventional benchmarks. Post-hoc comparisons demonstrated that the RE + EAA group exhibited a significantly greater increase in the ratio compared with both the CON (*p* < 0.001) and EAA groups (*p* < 0.05). The RE and EAA groups also showed moderate increases, although smaller than those observed in the RE + EAA group. Overall, the RE + EAA intervention produced the most pronounced improvement in the anabolic-to-catabolic balance, reflected by a 113.94% increase in the follistatin/myostatin ratio.

### 3.5. Change inflammatory cytokine factors

**Figure 5.**
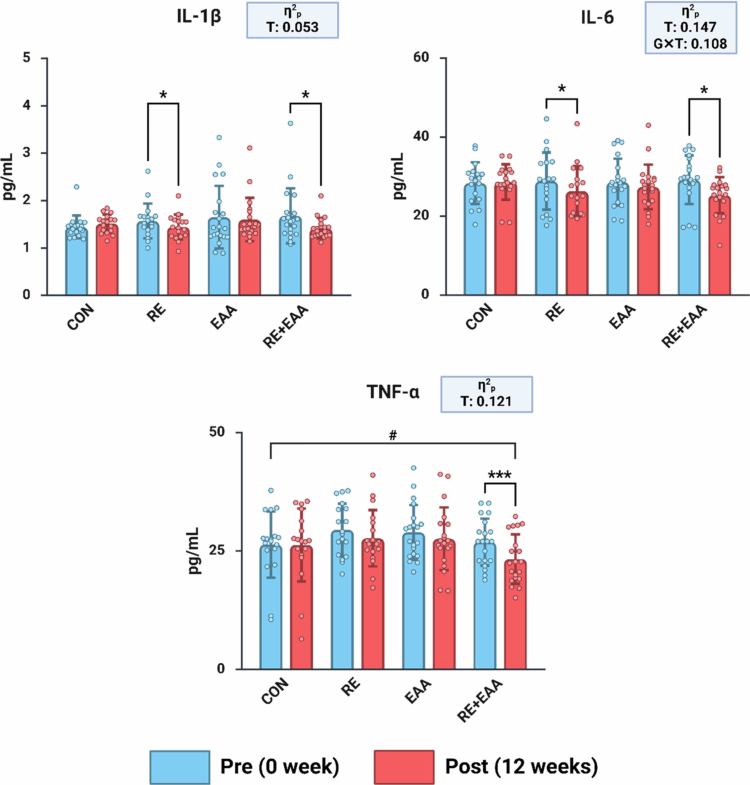
Alterations in inflammatory cytokine factors. Values are reported as Mean ± SD. CON, control group; RE, resistance exercise group; EAA, essential amino acid intake group; RE+EAA, combined resistance exercise and essential amino acid intake group. Data analysis was conducted with two-way repeated measures ANOVA. Interaction effect: ^#^*p* < 0.05, ^###^*p* < 0.001; paired t-test: *p < 0.05; ***p < 0.001. Effect sizes (η²p) were interpreted as small (0.01–0.059), medium (0.06–0.139), and large (≥0.14).

Figure 5 shows changes in inflammatory cytokine factor. Two-way repeated-measures ANOVA revealed a significant group-by-time interaction for IL-6 (F(3, 72) = 2.909, *p* = 0.040, partial η² = 0.108), and significant main effects of time for IL-6 (F(1, 72) = 12.444, *p* < 0.001, partial η² = 0.147), IL-1β (F(1, 72) = 3.992, *p* = 0.050, partial η² = 0.053), and TNF-*α* (F(1, 72) = 9.869, *p* = 0.002, partial η² = 0.121). Paired t-tests showed significant reductions in IL-1β after 12 weeks compared to baseline in both the RE group (*p* < 0.05) and the RE + EAA group (*p* < 0.05). Similarly, IL-6 levels were significantly reduced in both the RE (*p* < 0.05) and RE + EAA (*p* < 0.05) groups at 12 weeks. For TNF-*α*, a significant decrease was found only in the RE + EAA group (*p* < 0.001) after 12 weeks compared to baseline.

## 4. Discussion

This randomised controlled trial provides compelling evidence that a relatively short 12-week intervention combining RE with EAA supplementation elicits synergistic anabolic and anti-inflammatory effects in healthy older women. Unlike RE or EAA alone, the combined intervention not only increased skeletal muscle mass but also improved multiple indices of muscle function, including strength, agility, and cardiorespiratory endurance, highlighting its dual action on muscle quantity and quality. At the serum level, the intervention markedly suppressed myostatin while elevating follistatin and IL-15, resulting in an approximately 114% increase in the follistatin-to-myostatin ratio, a key indicator of anabolic activation. These systemic adaptations were accompanied by significant reductions in circulating IL-6, IL-1β, and TNF-*α*, underscoring that concurrent mechanical and nutritional stimuli effectively counteract age-related inflammatory stress. Collectively, these findings position the RE + EAA regimen as a potent, non-pharmacological strategy to simultaneously enhance muscle anabolism and mitigate inflammation in the aging population.

RE is well recognised as a primary intervention to prevent age-related muscle loss by activating the mTOR signaling pathway and stimulating satellite cells through mechanical loading, thereby enhancing MPS [25]. Similarly, supplementation with EAA, particularly those rich in leucine, promotes MPS by activating the mTOR and Sestrin-2/GATOR2 pathways [26], while concurrently suppressing the AMPK/FoxO axis to reduce muscle protein breakdown (MPB) [27]. However, older adults commonly experience a physiological condition known as anabolic resistance, characterised by a blunted muscle response to dietary protein or amino acid stimulation. Therefore, supplementation with potent anabolic agents such as leucine has been proposed as an effective strategy to overcome this limitation [28]. Previous studies have reported that either RE or EAA supplementation alone can positively influence muscle mass or muscle function in older women [29,30]. In contrast, the present study found that only the combined intervention of RE and EAA resulted in a significant increase in muscle mass, supporting a synergistic interaction between the two factors and suggesting that their combination may more effectively stimulate MPS and mitigate age-related muscle loss [29–31]. Conversely, some studies have shown increases in muscle mass following either RE or EAA supplementation alone [25,27]. Such discrepancies may be attributed to differences in participants’ health status, comorbidities, or protein metabolic capacity. For instance, studies involving participants with sarcopenia, cancer, or severe frailty have reported muscle mass gains with EAA supplementation alone [32]. However, since the participants in the present study were relatively healthy older women, a single intervention might not have provided sufficient anabolic stimulus to elicit substantial MPS. Moreover, considering the mean ages of the RE (72.89 ± 4.89 years) and EAA (72.8 ± 4.57 years) groups, the 12-week duration and intensity of the intervention may have been insufficient to fully overcome age-related anabolic resistance [33].

While EAA supplementation alone did not produce marked hypertrophic effects, its combination with RE appeared to synergistically potentiate molecular anabolic signaling. Among essential amino acids, leucine robustly activates the mTORC1 pathway via the Sestrin2–Rag GTPase complex, thereby enhancing translational efficiency and MPS independently of insulin signaling [20]. Previous studies have shown that post-exercise ingestion of 5–10 g of EAA can optimise muscle recovery and MPS in older adults [20], and in the present study, the RE + EAA group demonstrated the most pronounced improvements in IL-15 and in the follistatin-to-myostatin (F/M) ratio. These findings suggest that, even when macroscopic hypertrophy reaches a plateau, EAA supplementation may help maintain anabolic signalling sensitivity, thereby sustaining muscle quality.

Unlike the findings for muscle mass, the pattern of change observed in muscle fitness indicators differed. Although the RE group did not experience substantial muscle mass gain, significant enhancements in muscle strength, lower-limb function, cardiorespiratory endurance, and the TUG test, which assesses functional agility, were most pronounced in this group and were comparable to those observed in the combined intervention group. Additionally, significant improvements in balance ability were observed across all intervention groups. These data indicate that neuromuscular adaptations or improved functional efficiency from exercise training can manifest even without evident muscle hypertrophy, consistent with prior research [34].

Importantly, the group receiving both RE and EAA exhibited increases in muscle mass as well as enhancements in muscle fitness parameters. Collectively, these findings suggest that the combined intervention may have provided synergistic mechanical and nutritional stimuli, leading to parallel improvements of muscle quantity and function. Although all intervention groups except the control group demonstrated significant decreases in fat mass, the combined intervention group was the only cohort to achieve both muscle mass and function improvements simultaneously. Therefore, in healthy older adults, a combined intervention of RE and EAA may serve as a potential strategy, rather than a definitive clinical approach, for mitigating sarcopenia and supporting muscle function.

The observed increases in muscle mass and enhancements in muscle fitness within the RE + EAA group are likely strongly linked to alterations in myokine profiles, reflecting an integrated molecular response to both mechanical and nutritional stimuli. Consistent with this interpretation, the combined RE + EAA intervention resulted in decreased myostatin, increased follistatin, and elevated IL-15 levels, aligning with mechanistic evidence from previous clinical studies [14]. Myostatin promotes MPB by binding to the activin type IIB receptor (ActRIIB), activating Smad2/3 signaling, and suppressing MyoD expression [4,35], whereas follistatin counteracts this inhibition by blocking or sequestering myostatin, thereby stimulating MPS [36]. Clinical evidence has similarly shown that EAA supplementation can reduce myostatin and elevate follistatin in plasma and muscle tissue [37]. In the present study, the F/M ratio increased by 44.03% in the RE group, 29.80% in the EAA group, and 113.94% in the RE + EAA group, suggesting that mechanical and nutritional stimuli acted synergistically to enhance the anabolic environment. IL-15, a myokine abundantly expressed in skeletal muscle, further contributes to this process by activating Akt/mTOR signaling and suppressing TNF-*α* expression, thereby stimulating MPS and inhibiting MPB [38]. While the RE-only group showed no significant change in IL-15, the RE + EAA group exhibited a marked increase, implying that combined stimuli may be necessary to overcome age-related anabolic resistance and fully mobilize IL-15 expression in older adults [33,39]. Taken together, the simultaneous increase in IL-15 and follistatin, alongside a decrease in myostatin, indicates that the combined intervention likely created a more favourable anabolic milieu, mitigating catabolic and inflammatory signaling. Nevertheless, while these physiological interpretations are supported by established evidence, they should be viewed as indicative rather than definitive. Because the present mechanistic inferences are derived from systemic circulating markers rather than direct muscle biopsy data, the observed responses should be interpreted as reflecting broader systemic anabolic adaptations rather than conclusive intramuscular molecular changes.

The observed elevation of IL-15, together with reductions in myostatin and pro-inflammatory cytokines, suggests that the combined intervention helped establish a metabolically favourable environment within skeletal muscle. Chronic low-grade inflammation that accompanies aging, termed inflammaging, is a major contributor to sarcopenia, as cytokines such as TNF-*α*, IL-6, and IL-1β promote protein degradation and inhibit muscle regeneration [40,41]. In the present study, significant decreases in all three cytokines were observed only in the RE + EAA group, indicating that the combined intervention effectively alleviated systemic inflammatory stress. Although these changes may, in part, reflect the activation of muscle-derived anti-inflammatory signaling through IL-15 and related pathways, it is more plausible that they were driven predominantly by concurrent reductions in fat mass rather than direct muscle-specific mechanisms. Adipose tissue is a major source of pro-inflammatory cytokines, and even modest fat loss can substantially reduce circulating TNF-*α*, IL-6, and IL-1β in older adults [42]. Thus, the observed anti-inflammatory response likely reflects improvements in whole-body metabolic health mediated by reduced adipose-derived inflammation, which secondarily contributed to an enhanced anabolic environment in muscle.

Mechanistically, pro-inflammatory cytokines such as IL-1β, IL-6, and TNF-*α* activate the NF-κB, MAPK, and JAK/STAT pathways, amplifying inflammatory gene expression and disrupting muscle homeoostasis [43–45]. Accordingly, the suppression of these cytokines in the RE + EAA group likely indicates that improvements in adipose tissue metabolism attenuated these signalling cascades, restoring systemic metabolic balance and reducing inflammation. The pronounced decreases in TNF-*α*, IL-6, and IL-1β therefore appear to result from fat-loss-driven improvements in adipose–muscle cross-talk, rather than from muscle-intrinsic signaling alone.

Previous clinical trials have also shown that reductions in body fat are closely associated with declines in systemic inflammation and improvements in metabolic health among older adults [46]. In contrast, our study extends these findings by demonstrating that the RE + EAA combination not only reduced inflammatory cytokines but also concurrently improved muscle-related markers such as IL-15 and the follistatin/myostatin ratio, suggesting a coordinated adaptation between muscle and adipose tissue. Together, these findings support the interpretation that the combined intervention mitigated inflammaging primarily through fat-loss–mediated anti-inflammatory effects, which, in turn, enhanced muscle anabolic sensitivity and metabolic homeostasis in healthy older women [47].

Despite the physiological and clinical significance, this study is subject to several limitations. First, although participants were instructed to maintain consistent dietary intake throughout the intervention, complete control over daily macronutrient composition and habitual protein intake was not achieved. Because total dietary protein intake strongly influences muscle protein synthesis, unmonitored variability in background nutrition could have contributed to individual differences in muscle-related outcomes [48]. Second, muscle mass was estimated using bioelectrical impedance analysis (BIA), which reflects total lean body mass rather than isolated skeletal muscle tissue. Accordingly, the reported changes in “muscle mass” may partially represent alterations in total lean tissue or hydration status rather than direct skeletal muscle hypertrophy, particularly in older adults, where fluid balance can vary [49]. Third, the intervention period was limited to 12 weeks, which may have been insufficient to capture long-term adaptations. Fourth, participants exhibited relatively high baseline levels of physical function, which may have limited the sensitivity of some functional outcome measures due to potential ceiling effects. Fifth, muscle status was evaluated using indirect biomarkers such as serum myokines and inflammatory cytokines, without the use of direct markers of muscle protein metabolism, including muscle biopsy, amino acid oxidation rate, or nitrogen balance. Future research should utilise more direct physiological assessments to strengthen evaluation of intervention outcomes. Finally, myokine and inflammatory marker levels were measured at only two time points, prior to and following the intervention, making it challenging to assess the progression of physiological changes or to distinguish transient from sustained effects. Subsequent studies should include intermediate and follow-up assessments with time-series analysis during the intervention to capture these dynamics.

## 5. Conclusion

This study evaluated the effects of 12 weeks of RE, EAA, and their combination on muscle mass, muscle fitness, myokines, and inflammatory markers in insulin resistance free women aged ≥ 65. Given the elderly’s susceptibility to insulin resistance and metabolic complications, this study is notable for its focus on early intervention before pathological onset. Combined RE and EAA was more effective than the either intervention alone in enhancing muscle mass and muscle fitness, key myokines (myostatin, follistatin, and IL-15), and reducing inflammatory cytokines (IL-1β, IL-6, and TNF-*α*). These findings indicate that, for metabolically healthy older women, the combined strategy can be a more potent non-pharmacological option for preventing sarcopenia and promoting physical function. Moreover, given age-related anabolic resistance, the dual intervention may offer broader and more sustained benefits than either strategy alone. Additionally, this study highlights the potential of multimodal, non-pharmacological approaches for early sarcopenia prevention, and provides a basis for personalized exercise-nutrition prescriptions.

## Data Availability

The data supporting the findings of this study are available from the corresponding author upon reasonable request.
